# Organizational determinants of patients' experiences of care for breast, lung and colorectal cancers

**DOI:** 10.1111/j.1365-2354.2008.00966.x

**Published:** 2009-05

**Authors:** M McCARTHY, P DATTA, C SHERLAW-JOHNSON

**Affiliations:** UCL Department of Epidemiology and Public Health, University College LondonLondon; UCL Department of Mathematics, University College LondonLondon, UK

**Keywords:** patient satisfaction, cancer, service standards, health service management, hospitals

## Abstract

Organizational characteristics in English NHS hospitals and the experiences of patients with three common cancers – breast, colorectal and lung – were examined using secondary data analyses. Two specific measures of satisfaction, *Respect and Dignity*, reflecting inpatient care, and *Communication* reflecting hospital outpatient care, were drawn from a national survey of cancer patients after first hospital treatment. They were compared at hospital level with hospital cancer service standards, and measures of hospital provision, each drawn from national surveys. *Respect and Dignity* was greater in hospitals with fewer complaints, slower admission procedures and a greater proportion of medicine consultants, for breast and colorectal cancers only. For breast cancer alone, *Respect and Dignity* was greater in hospitals achieving more participation in meetings by lead team members at the cancer unit level. For lung cancer alone, there were tumour-specific team organizational measures (relating to outpatient assessment) associated with *Communication*. However, the majority of recorded standards did not show associations, and there were occasional negative associations (dissatisfaction). The impact of organizational factors on patients may be examined through observational studies when experimental designs are not possible. Understanding how organizational factors affect quality of care for cancer patients can contribute to planning and management of cancer services.

## INTRODUCTION

There is strong professional and political concern to promote patient-centred health care (Coulter & Fitzpatrick 2000; Department of Health 2005; Kennedy 2006). While quality of life is frequently assessed for patients in cancer treatment clinical trials, patients' experience of their care is rarely recorded, while satisfaction with routine cancer care, either outpatient (Thomas *et al.* 1997) or inpatient (Skarstein *et al.* 2002), has only occasionally been reported. A systematic review by Crow *et al.* (2002) described the individual level determinants of patient satisfaction (for all diseases), including health status, age, ethnicity, expectations, prior experience, information-giving and choice of service. In a study of 2239 patients after inpatient episodes (all diseases), Jenkinson *et al.* (2002) found that ‘age and overall self-assessment were only weakly associated … the major determinants of patient satisfaction were physical comfort, emotional support and respect for patient preferences’. In palliative care, a national post-bereavement survey (Fakhoury *et al.* 1996) found that satisfaction with nursing and medical staff was strongly associated with the frequency of attendance and less strongly with the characteristics of the individual or the carer.

Several countries in recent years, including UK (Department of Health 2000a), France (République Française 2002), USA (Institute of Medicine 1999) and Australia (Department of Health and Ageing 2005), have developed national cancer service plans, which seek to put the patient at the centre of an improved organizational structure. Will this improve patient satisfaction? We have investigated how the data from the patients' survey are related to measures of cancer standards and service facilities – at hospital level. The study compares dimensions of satisfaction from a national survey of cancer patients with data about the same hospitals drawn from other national cancer datasets recorded at the same time (McCarthy *et al.* 2007).

## METHODS

The survey of cancer patients in England in 2000–1 was one of a series of surveys to support the increased orientation of the NHS towards the patient's perspective. The survey drew a sample of patients discharged from hospitals with a diagnosis of one of six common cancers, which included breast, colorectal and lung (Department of Health 2004a). Of 135 000 questionnaires issued by the hospitals, there were 65 337 respondents from 172 hospital trusts in England (35 674 responses for the three cancers considered here). The survey questionnaire asked over 60 questions, and factor analysis was used by the survey authors to reduce these to 10 dimensions, represented by a single question, across the patient pathway. We examined two contrasting questions: Question D15 ‘Time spent on explaining condition of first visit’, which is used to represent outpatient clinician–patient *Communication*, and Question B7 ‘Doctor not treated with respect and dignity by doctor-nurse’ which is a valuation of *Respect and Dignity* for in-hospital care. While the data were recorded at individual level, we averaged the responses of patients at hospital level to link their experiences to the services.

The Manual for Cancer Services was developed by the English Department of Health. It was based upon the principles and recommendations of a Report by the Chief Medical Officer (Department of Health 1995), tumour-specific ‘Improving Outcomes Guidance’ (Department of Health, Clinical Outcomes Group, Cancer Guidance sub-group 1996, 1997, 1998), and advice from managers in the Trent Region (Department of Health 2004b). The Manual gave guidance across 10 areas of the patient pathway – from referral and diagnosis, investigation and treatment, to continuing and palliative care – and these were used to create 180 cancer standards, divided into broad themes (see Table 1). Each standard was defined and the expected evidence stated in more detail. In 2001, peer review teams visited hospitals to measure these standards for existing cancer services. The data were held by the Department of Health and local data were returned to individual trusts. To create prior hypotheses and for statistical reasons, we selected a limited number of items that reflected the general aspects of hospital services, cancer centre and unit services and (for breast, colorectal and lung cancers only) aspects related to specific tumour types (Table 1).

**Table 1:** Main headings of cancer standards (Department of Health 2000b), and numbers used in analysis

Standard title	Total available	Number used in analysis
Patient-centred care	5	0
Specialist multi-disciplinary teams		
Breast	39	15
Colorectal	35	13
Lung	36	14
Diagnostic services – pathology	7	0
Provision of Non-surgical Oncology to Cancer Units	5	0
Radiotherapy	60	1 (% summed total)
Chemotherapy	45	1 (% summed total)
Specialist Palliative Care Services	11	0
Education, Training & Continuous Professional Development	2	0
Communication between Primary, Secondary and Tertiary sectors	3	0
Coordination and organization of cancer units	15	15

The Audit Commission collected data in 2000 on hospital staff and facilities, which has contributed to the Acute Hospital Portfolio now held by the Healthcare Commission (Healthcare Commission 2006). We gained the data directly from the (then) Audit Commission as they are not formally in the public domain. The data covered a range of domains (see Table 2), of which we selected a small number of specific variables which (1) had completeness above 80%; (2) we considered related to patient outcomes.

**Table 2:** Data domains drawn from Acute Hospital Portfolio, and (bold) chosen for the analysis

Ward staffing data
Total Finished Consultant Episodes (FCEs) – year
Clinical nurse specialists, whole time equivalents (WTEs)
Pressure sores – incidence %
Standardized ward patient accidents per 100 available beds
**All formal complaints per 1000 FCEs**
Relative WTEs per bed, Relative cost per WTE
Day surgery units data
Total staffed beds and chairs
Total day cases – month
Day cases per staffed bed or chair per month
Weighted throughput per staffed bed or chair per month
Day cases per 1000 admissions
Total staff (WTE) per bed or chair per month
Accident and Emergency data
**Total attendances in a year**
**% of patients admitted within 4 h**
**Doctor WTE per attendances**
**Nurse WTE per attendances**
Medicines Management data
British National Formulary spend for malignant diseases/immuno-suppression, per FCE
British National Formulary spend: treatment of infections per FCE
Medical staffing data
**Ratio of outpatients**
**Consultant WTE per admissions**
**Anaesthetist Consultant WTE per 1000 admissions**
**Medicine Consultant WTE per 1000 admissions**
**Pathology Consultant WTE per 1000 admissions**
**Radiology Consultant WTE per 1000 admissions**
Radiology data
Radiology examinations, outpatients and inpatients
Computerized Tomography (CT), barium enemas
Total number of radiographers per 1000 admissions
Waiting times for symptomatic mammography,
Waiting times for nuclear medicine,
Waiting times for CT,
Waiting times for Magnetic Resonance Imaging
% of total examinations unreported
% of exams reported by radiologist
Ratio of inpatient exams to activity (per FCE)
Ratio of outpatients exams to activity (per outpatient visit)
Information technology and digital technology score
Rapid access clinics – breast
Rapid access clinics – colorectal

We linked the data by hospital of treatment by using codes provided by the Department of Health. This required some attention because of the range of names of hospitals and the need to allocate them accurately to changing configurations. Where necessary, we amalgamated trust results so that they reflected the changing configurations. We made statistical tests using Spearman rank correlation where both variables were continuous (for the patient survey and the acute hospital portfolio) and point-biserial correlation when one variable was a dichotomous variable (cancer standards) and the other a continuous variable.

## RESULTS

### Acute hospital portfolio (Table 3)

For both breast and colorectal cancers, but not lung cancer, there were significant associations between *Respect and Dignity* and hospitals with fewer complaints, slower admissions and a larger number of medicine consultants. Hospital attendances were associated for colorectal cancer, and a higher proportion of outpatients to admissions for breast cancer. However, overall doctor and nurse staffing levels, and support medical specialists showed no association for any of the cancer types. The second satisfaction measure, *Communication*, was not associated with these measures for any of the cancer types.

**Table 3:** Association (rank correlations) between measures from Acute Hospital Portfolio and two measures of satisfaction for patients with breast, colorectal and lung cancers

	Breast		Colorectal		Lung	
Variables	Respect and Dignity	Communication	Respect and Dignity	Communication	Respect and Dignity	Communication
Formal complaints per 1000 FCEs	0.22**	−0.03	0.21**	0.13	0.10	0.10
	*P* = 0.01	*P* = 0.75	*P* = 0.01	*P* = 0.14	*P* = 0.25	*P* = 0.24
Total attendances in a year	0.11	−0.07	0.26**	−0.12	0.02	−0.08
	*P* = 0.18	*P* = 0.38	*P* = 0.002	*P* = 0.15	*P* = 0.78	*P* = 0.35
% of patients admitted ≤ 4 h	−0.26**	0.10	−0.35**	−0.132	−0.102	−0.008
	*P* = 0.003	*P* = 0.23	*P* < 0.001	*P* = 0.13	*P* = 0.24	*P* = 0.93
Doctor WTE per attendances	−0.03	0.06	−0.04	−0.11	0.09	0.05
	*P* = 0.71	*P* = 0.49	*P* = 0.64	*P* = 0.1	*P* = 0.31	*P* = 0.57
Nurse WTE per attendances	0.03	−0.22	0.05	0.02	0.15	0.09
	*P* = 0.71	*P* = 0.13	*P* = 0.54	*P* = 0.77	*P* = 0.06	*P* = 0.28
Ratio of outpatients to admissions	0.23**	−0.11	0.130	0.05	0.07	0.01
	*P* = 0.008	*P* = 0.19	*P* = 0.14	*P* = 0.56	*P* = 0.41	*P* = 0.90
Consultant WTE per 1000 admissions	0.17	−0.07	0.07	0.02	0.12	−0.05
	*P* = 0.05	*P* = 0.41	*P* = 0.43	*P* = 0.79	*P* = 0.15	*P* = 0.59
Anaesthetist consultant WTE per 1000 admissions	0.10	−0.01	−0.08	−0.1	0.02	0.03
	*P* = 0.25	*P* = 0.89	*P* = 0.35	*P* = 0.26	*P* = 0.79	*P* = 0.68
Medicine consultant WTE per 1000 admissions	0.21**	−0.12	0.19*	0.093	0.16	−0.04
	*P* = 0.01	*P* = 0.17	*P* = 0.03	*P* = 0.28	*P* = 0.07	*P* = 0.62
Pathology consultant WTE per 1000 admissions	0.14	−0.03	0.05	−0.10	0.12	−0.11
	*P* = 0.11	*P* = 0.69	*P* = 0.59	*P* = 0.26	*P* = 0.17	*P* = 0.19
Radiology consultant WTE per 1000 admissions	0.14	−0.11	−0.01	0.07	0.05	−0.07
	*P* = 0.11	*P* = 0.19	*P* = 0.94	*P* = 0.40	*P* = 0.56	*P* = 0.44

* Correlation significant at the 0.05 level (two-tailed)

** Correlation significant at the 0.01 level (two-tailed).

FCE, Finished Consultant Episode; WTE, whole time equivalent.

### Cancer service standards

#### At hospital level (Table 4)

There were significant associations for five breast cancer standards with the *Respect and Dignity* measure of patient experience. These standards were related to members of the cancer unit group (lead clinician, lead nurse, lead manager). There were no associations with *Respect and Dignity* for either colorectal or lung cancers. Greater dissatisfaction with *Communication* was associated with two standards for breast cancer, whereas no significant associations were found for the other two tumour types with the measure of *Communication*.

**Table 4:** Associations (rank correlations and point biserial) between Cancer Standards for hospital cancer centres and units and two measures of satisfaction measures for patients with breast, colorectal and lung cancers

	Breast		Colorectal		Lung	
Variables	Respect and Dignity	Communication	Respect and Dignity	Communication	Respect and Dignity	Communication
10.2/1 – Referral guidelines for cancer sites not covered by the unit MDT	−0.07	0.04	−0.16	−0.09	−0.05	−0.02
	*P* = 0.47	*P* = 0.67	*P* = 0.08	*P* = 0.35	*P* = 0.60	*P* = 0.83
10.2/2 – Cancer services Lead Clinician for the cancer unit	−0.20*	0.16	−0.07	−0.08	−0.16	0.08
	*P* = 0.03	*P* = 0.08	*P* = 0.45	*P* = 0.40	*P* = 0.09	*P* = 0.36
10.2/3 – Written job description for the lead role	−0.15	0.27**	0.04	−0.14	0.07	−0.10
	*P* = 0.10	*P* = 0.003	*P* = 0.63	*P* = 0.13	*P* = 0.44	*P* = 0.29
10.2/4 – Specification of time available and administrative support for the Lead Clinician	−0.09	0.26**	0.05	0.02	0.07	−0.09
	*P* = 0.33	*P* = 0.004	*P* = 0.60	*P* = 0.84	*P* = 0.44	*P* = 0.35
10.2/5 – Regular review of time/support available to lead Clinician	−0.09	0.14	0.008	0.03	0.06	−0.06
	*P* = 0.31	*P* = 0.14	*P* = 0.93	*P* = 0.75	*P* = 0.53	*P* = 0.47
10.2/6 – Lead Clinician member of cancer unit group	−0.22*	0.18	−0.06	−0.02	−0.08	−0.01
	*P* = 0.02	*P* = 0.05	*P* = 0.53	*P* = 0.84	*P* = 0.39	*P* = 0.90
10.2/7 – Cancer services lead nurse for the cancer unit	−0.17	0.06	−0.07	−0.008	0.002	−0.16
	*P* = 0.05	*P* = 0.54	*P* = 0.43	*P* = 0.93	*P* = 0.98	*P* = 0.09
10.2/8 – Written job description for the lead nurse	−0.18*	0.16	−0.06	0.03	−0.03	−0.09
	*P* = 0.04	*P* = 0.08	*P* = 0.53	*P* = 0.73	*P* = 0.75	*P* = 0.31
10.2/9 – Cancer services lead manager for the cancer unit	−0.24**	0.07	−0.06	−0.01	−0.04	0.16
	*P* = 0.007	*P* = 0.41	*P* = 0.51	*P* = 0.90	*P* = 0.69	*P* = 0.07
10.2/10 – Written job description for the lead manager	−0.15	0.16	−0.02	−0.04	−0.01	−0.08
	*P* = 0.09	*P* = 0.07	*P* = 0.80	*P* = 0.69	*P* = 0.89	*P* = 0.38
10.2/11 – Named cancer unit group with membership	−0.23*	0.14	−0.05	−0.03	−0.01	0.01
	*P* = 0.01	*P* = 0.13	*P* = 0.56	*P* = 0.75	*P* = 0.88	*P* = 0.89
10.2/12 – Terms of reference for the group	−0.15	−0.020	−0.009	−0.05	−0.06	−0.009
	*P* = 0.10	*P* = 0.83	*P* = 0.92	*P* = 0.56	*P* = 0.53	*P* = 0.92
10.2/13 – Primary care representation on the group	−0.09	0.15	−0.003	−0.12	0.02	0.05
	*P* = 0.33	*P* = 0.09	*P* = 0.98	*P* = 0.17	*P* = 0.82	*P* = 0.55
10.2/14 – Named cancer site leads for each cancer site	−0.17	0.09	−0.15	0.02	−0.10	−0.13
	*P* = 0.06	*P* = 0.33	*P* = 0.11	*P* = 0.79	*P* = 0.286	*P* = 0.16
10.2/15 – Reporting requirements to the cancer registry	−0.15	0.18	−0.008	−0.07	−0.02	−0.09
	*P* = 0.10	*P* = 0.05	*P* = 0.93	*P* = 0.45	*P* = 0.82	*P* = 0.33

* Correlation significant at the 0.05 level (two-tailed);

** Correlation significant at the 0.01 level (two-tailed).

#### Tumour-specific services (Table 5)

Four team organizational measures for lung cancer were all associated with greater satisfaction with the *Communication* measure: these were frequent meetings, operational policy meetings, policy on communicating with GP and policy for urgent referrals. For breast cancer, there was a single association between operational policy meetings and *Respect and Dignity*, and for a named team lead with *Communication*. Colorectal cancer showed only an association with Lead clinician written responsibilities and *Respect and Dignity*. No associations were found for any tumour type with the presence in the team of other lead clinicians, in imaging or histopathology (data not shown).

**Table 5:** Associations (rank correlation and point biserial) between Cancer Standards for tumour-specific teams and two measures of patient satisfaction for patients with breast, colorectal and lung cancers

	Breast		Colorectal		Lungy	
Standard	Respect and Dignity	Communication	Respect and Dignity	Communication	Respect and Dignity	Communication
Team structure						
Named team lead	0.10	−0.22*	0.12	0.15	0.069	−0.03
	*P* = 0.26	*P* = 0.02	*P* = 0.18	*P* = 0.11	*P* = 0.45	*P* = 0.71
Lead clinician written responsibilities	0.12	−0.13	0.18*	0.135	−0.02	0.01
	*P* = 0.18	*P* = 0.16	*P* = 0.04	*P* = 0.139	*P* = 0.85	*P* = 0.91
Team meetings						
Core members attendance at team meetings	−0.04	0.12	0.04	0.09	−0.05	−0.18*
	*P* = 0.64	*P* = 0.19	*P* = 0.63	*P* = 0.34	*P* = 0.56	*P* = 0.04
Operational policies						
Operational policy meetings	−0.20*	−0.10	0.002	0.03	0.12	−0.20*
	*P* = 0.03	*P* = 0.30	*P* = 0.99	*P* = 0.77	*P* = 0.20	*P* = 0.03
Written operational policy – communication of diagnosis with GP	0.02	−0.02	0.011	−0.020	0.02	−0.23**
	*P* = 0.80	*P* = 0.84	*P* = 0.907	*P* = 0.824	*P* = 0.79	*P* = 0.01
Written operational policy – provision of information to GP on urgent referrals	−0.08	−0.05	0.038	0.006	−0.02	−0.22*
	*P* = 0.37	*P* = 0.62	*P* = 0.676	*P* = 0.945	*P* = 0.79	*P* = 0.02
Breast Surgeon						
Designated sessions	0.26**	−0.08				
	*P* = 0.005	*P* = 0.38				
Accredited British Association Surgical Oncology	0.09	0.21*				
	*P* = 0.41	*P* = 0.04				
Nurse specialist qualification						
Registered	−0.11	−0.179	−0.14	0.06	0.008	−0.04
	*P* = 0.29	*P* = 0.09	*P* = 0.18	*P* = 0.53	*P* = 0.94	*P* = 0.67
Obtained	−0.05	−0.159	0.11	−0.06	0.01	−0.20*
	*P* = 0.65	*P* = 0.12	*P* = 0.26	*P* = 0.53	*P* = 0.89	*P* = 0.04
Degree	0.02	0.092	−0.28**	−0.04	0.14	−0.04
	*P* = 0.87	*P* = 0.37	*P* = 0.005	*P* = 0.71	*P* = 0.18	*P* = 0.67
Patient centred care						
Written information material available	−0.22*	−0.16	0.06	0.04	−0.16	−0.04
	*P* = 0.01	*P* = 0.09	*P* = 0.52	*P* = 0.65	*P* = 0.08	*P* = 0.66
Guidelines						
Referral guidelines for the cancer site	0.22*	0.101	−0.01	−0.07	−0.04	−0.02
	*P* = 0.02	*P* = 0.28	*P* = 0.91	*P* = 0.41	*P* = 0.64	*P* = 0.79
Data collection						
Network-wide dataset	0.12	0.20*	0.01	0.02	0.23**	0.01
	*P* = 0.21	*P* = 0.03	*P* = 0.90	*P* = 0.79	*P* = 0.009	*P* = 0.87
Recording data for individual patients	0.20*	0.004	0.10	0.05	0.24**	0.17
	*P* = 0.04	*P* = 0.97	*P* = 0.33	*P* = 0.61	*P* = 0.008	*P* = 0.06

* Correlation significant at the 0.05 level (two-tailed);

** Correlation significant at the 0.01 level (two-tailed).

Contrary to the expected trend, the presence of designated sessions for a specialist breast surgeon was associated with greater dissatisfaction with *Respect and Dignity*, and a surgeon accredited with the British Association of Surgical Oncology (BASO) with *Communication* dissatisfaction.

The standards included three measures of nurse specialist qualifications: none was associated for breast cancer, and one each only for colorectal cancer (for *Respect and Dignity*) and lung cancer (for *Communication*).

The single cancer standard ‘Written information available’ was associated with *Respect and Dignity* for breast cancer, but not otherwise for colorectal or lung. For three measures of undertaking patient satisfaction surveys within the Standards, none was associated with any of the three tumour types. Greater dissatisfaction was found for breast cancer with collection of a network-wide data set and *Communication*, for breast cancer with recording data for individual patients and *Respect and Dignity*, and for lung cancer with recording data for individual patients and *Communication*.

## DISCUSSION

Significant statistical associations were found between hospital organizational characteristics and measures of patient experiences, in expected directions. *Respect and Dignity* (a measure of satisfaction with inpatient care) showed stronger associations at hospital level than within tumour-specific services, while *Communication* (a measure of the initial outpatient appointment) only showed associations at tumour-service level. There was greater satisfaction with *Respect and Dignity* at hospitals with fewer formally recorded complaints; it was also greater in hospitals with longer average admission times, but this may reflect more detailed investigation and higher standards in the admission period.

There were some associations of the standard for the presence of medical staff leads within the cancer group for *Respect and Dignity* for breast cancer and for *Communication* with colorectal cancer. Clinical team performance, assessed by team members, has been associated with team inputs including leadership (Wagner *et al.* 2001; Haward *et al.* 2003; Shortell *et al.* 2004), but not previously associated with patient satisfaction. However, most other of the other cancer standards, which had been set by professional opinion (Department of Health 2004b) and regarded as indicators of system performance, were not associated with either of the satisfaction measures. This may be low validity – that the measures don't reflect patient-orientation – or lack of effect – that the services measured do not influence patient experience. But if these measures are unlikely to influence patient experience, what should be measured?

A methodological strength of our study is that we drew on three independent sets of data relating to hospitals relating to a similar period of time. Only a few of the associations reached a *P-*value of <0.01: it is possible that some of the associations were by chance, but there could also have been chance false-negatives. We used a limited number of statistical comparisons, with prior hypotheses, reducing interpretation problems of multiple testing. We used non-parametric tests for some comparisons, which do not require normally-distributed data, and drew on rankings. A limitation included the need to group the patients together by hospital trust: the hospital characteristics were not individually linked to those experienced by the surveyed patients.

While medical evidence for cancer treatment has accumulated over decades, scientific evidence for the organizational structures patients prefer is much less well-developed. Randomized trials are unlikely to be undertaken, as statistical power would need large numbers of participating hospitals (the unit of analysis (Rychetnik *et al.* 2002) rather than collecting from individual patients). There is increasing focus on capture of patient-level information through electronic systems, but much less attention to recording hospital characteristics. More hospitals (78%) expect to influence clinician behaviour by improving clinical outcomes, but only 36% expect to improve patient satisfaction (Wallace *et al.* 2001). And Freeman and Walshe (2004, 335) found that, in England, while ‘structures and systems for clinical governance are well established … there is more perceived progress in areas concerned with quality assurance than quality improvement’.

Our study makes start to explaining cancer patient satisfaction with care through routinely available data. The associations are not strong, but suggest that at least some characteristics of hospitals may be reflected in patient satisfaction measures. Further work should seek to include differences in disease stage and treatment as explanatory factors, and to make more detailed investigations into differences in satisfaction between patient groups experiencing otherwise similar services.

## References

[b1] Coulter A, Fitzpatrick R, Albrecht GL, Fitzpatrick R, Scrimshaw RC (2000). The patient's perspective regarding appropriate health care. The Handbook of Social Studies in Health and Medicine.

[b2] Crow R, Gage H, Hampson S, Hart J, Kimber A, Storey L, Thomas H (2002). The measurement of satisfaction with health care: implications for practice from a systematic review of the literature. Health Technology Assessment.

[b3] Department of Health (1995). A Policy Framework for Commissioning Cancer Services: Report by the Expert Advisory Group: Guidance for Purchasers and Providers of Cancer Services.

[b4] Department of Health (2000a). The NHS Cancer Plan.

[b5] Department of Health (2000b). Manual of Cancer Services Standards.

[b6] Department of Health (2004a). National Survey of NHS Patients–Cancer: Analysis of Themes.

[b7] Department of Health (2004b). Manual of Cancer Standards.

[b8] Department of Health (2005). Creating a Patient-led NHS: Delivering the NHS Improvement Plan.

[b9] Department of Health and Ageing (2005). National Service Improvement Framework for Cancer.

[b10] Department of Health, Clinical Outcomes Group, Cancer Guidance sub-group (1996). Guidance on Commissioning Cancer Services: Improving Outcomes in Breast Cancer – the Research Evidence.

[b11] Department of Health, Clinical Outcomes Group, Cancer Guidance sub-group (1997). Guidance on Commissioning Cancer Services: Improving Outcomes in Colorectal Cancer – the Research Evidence.

[b12] Department of Health, Clinical Outcomes Group, Cancer Guidance sub-group (1998). Guidance on Commissioning Cancer Services: Improving Outcomes in Lung Cancer– the Research Evidence.

[b13] Fakhoury W, Addington-Hall J, McCarthy M (1996). Determinants of informal caregivers' satisfaction with services for dying cancer patients. Social Science and Medicine.

[b14] Freeman T, Walshe K (2004). Achieving progress through clinical governance? A national study of health care managers' perceptions in the NHS in England. Quality and Safety in Health Care.

[b15] Haward R, Amir Z, Borrill C, Dawson J, Scully J, West M, Sainsbury R (2003). Breast cancer teams: the impact of constitution, new cancer workload, and methods of operation on their effectiveness. British Journal of Cancer.

[b16] Healthcare Commission Acute Hospital Portfolio *[Internet]*.

[b17] Institute of Medicine (1999). Ensuring Quality Cancer Care.

[b18] Jenkinson C, Coulter A, Bruster S, Richards N, Chandola T (2002). Patients' experiences and satisfaction with health care: results of a questionnaire study of specific aspects of care. Quality and Safety in Health Care.

[b19] Kennedy I (2006). Learning from Bristol: Are We?.

[b20] McCarthy M, Gonzalez-Izquierdo A, Sherlaw-Johnson C, Khachatryan A, Coleman MP, Rachet B (2007). Comparative indicators for cancer network management in England: availability, characteristics and presentation. BMC Health Services Research.

[b21] République Française Plan Cancer 2003–2007 *[Intenet*.

[b22] Rychetnik L, Frommer M, Hawe P, Shiell A (2002). Criteria for evaluating evidence on public health interventions. Journal of Epidemiology and Community Health.

[b23] Shortell SM, Marsteller JA, Lin M, Pearson ML, Wu SY, Mendel P, Cretin S, Rosen M (2004). The role of perceived team effectiveness in improving chronic illness care. Medical Care.

[b24] Skarstein J, Dahl AA, Laading J, Fossa SD (2002). Patient satisfaction in hospitalized cancer patients. Acta Oncologica.

[b25] Thomas S, Glynne-Jones R, Chait I (1997). Is it worth the wait? A survey of patients' satisfaction with an oncology outpatient clinic. European Journal of Cancer Care.

[b26] Wagner EH, Austin BT, Davis C, Hindmarsh M, Schaefer J, Bonomi A (2001). Improving chronic illness care: translating evidence into action. Health Affairs (Millwood).

[b27] Wallace LM, Freeman T, Latham L, Walshe K, Spurgeon P (2001). Organisational strategies for changing clinical practice: how trusts are meeting the challenges of clinical governance. Quality in Health Care.

